# Some inequalities for $(p,q)$-mixed volume

**DOI:** 10.1186/s13660-018-1836-2

**Published:** 2018-09-19

**Authors:** Bin Chen, Weidong Wang

**Affiliations:** 1 Department of Mathematics, China Three Gorges University, Yichang, China; 2 Three Gorges Mathematical Research Center, China Three Gorges University, Yichang, China

**Keywords:** 52A20, 52A40, 52A39, $(p,q)$-mixed volume, Cyclic inequality, Monotonic inequality, Product inequality

## Abstract

Lutwak, Yang, and Zhang introduced the concept of $(p,q)$-mixed volume whose special cases contain the $L_{p}$-mixed volume and the $L_{p}$-dual mixed volume. In this article, associated with the $(p,q)$-mixed volumes, we establish related cyclic inequalities, monotonic inequalities, and product inequalities.

## Introduction and main results

At the end of the nineteenth century, Brunn and Minkowski pioneered the classical Brunn–Minkowski theory of convex bodies, which is the product of Minkowski linear combination of vectors and volumes in the Euclidean space. The core of this theory are mixed volume, mixed area measure, and the basic Brunn–Minkowski inequality. In recent years, Brunn–Minkowski theory attracted wide attention (see [1, 2]).

By the 1960s, Firey put forward the concept of $L_{p}$-Minkowski combination of convex bodies (see [2]). In 1993, Lutwak [3] introduced the $L_{p}$-Minkowski linear combination of convex bodies to the classical Brunn–Minkowski theory, proposed the notions of $L_{p}$-mixed volume, $L_{p}$-mixed quermassintegrals, and $L_{p}$-surface area measure, and obtained the corresponding integral expression, which extended the classical Brunn–Minkowski theory to $L_{p}$ space (called the $L_{p}$ Brunn–Minkowski theory). This new theory has attracted a large number of researchers’ interests in recent years (see [4–22]). Especially, the concept of $L_{p}$-mixed volume ($p\geq1$) plays an important role in $L_{p}$ Brunn–Minkowski theory (see [3, 23]).

The classical dual Brunn–Minkowski theory of star bodies was introduced by Lutwak [24] in 1975. In 1996, on the basis of $L_{p}$ harmonic radial combination, Lutwak [23] put forward the concept of $L_{p}$-dual mixed volume ($p\geq1$) and gave its integral expression. This means that the preliminary $L_{p}$ dual Brunn–Minkowski theory has been established. Afterwards, Grinberg and Zhang defined the notion of $L_{p}$ radial combination ($p>0$). In 2002, Gardner improved $p>0$ to $p\neq0$ in $L_{p}$ radial combination, and got a more extensive class of $L_{p}$-dual mixed volume ($p\neq0$). For more information about the classical dual Brunn–Minkowski theory and $L_{p}$ dual Brunn–Minkowski theory, please refer to [25–35].

Very recently, Huang et al. [10] constructed the dual curvature measure in dual Brunn–Minkowski theory. These measures are dual to Federer’s curvature measures which are fundamental in the classical Brunn–Minkowski theory. In 2018, Lutwak, Yang, and Zhang [36] made further work and introduced $L_{p}$ dual curvature measures which include $L_{p}$ surface area measure, dual curvature measures, and $L_{p}$ integral curvatures. Using this new concept, they introduced the $(p,q)$-mixed volume, which unifies $L_{p}$-mixed volume and $L_{p}$-dual mixed volume. Thus, $L_{p}$ Brunn–Minkowski theory and $L_{p}$ dual Brunn–Minkowski theory are partially unified.

Let *K* be a convex body if *K* is a compact, convex subset in an *n*-dimensional Euclidean space $\mathbb{R}^{n}$ with nonempty interior. The set of all convex bodies in $\mathbb{R}^{n}$ is written as $\mathcal {K}^{n}$. Let $\mathcal{K}_{o}^{n}$ denote the set of convex bodies containing the origin in their interiors. Let $\mathcal{S}_{o}^{n}$ denote the set of star bodies (about the origin) in $\mathbb{R}^{n}$. We write *u* for the unit vector and *B* for the unit ball centered at the origin, the surface of *B* denoted by $S^{n-1}$. We shall use $V(K)$ for the *n*-dimensional volume of the body *K* in $\mathbb{R}^{n}$.

Suppose that $\mathbb{R}$ is the set of real numbers. If $E\in\mathcal {K}^{n}$, the support function of *E*, $h_{E}=h(E,\cdot)$: $\mathbb {R}^{n}\rightarrow\mathbb{R}$, is defined by (see [1, 2])

$$h(E,x)=\max\{x\cdot y:y\in E\},\quad x\in\mathbb{R}^{n}, $$

where $x\cdot y$ denotes the standard inner product of *x* and *y* in $\mathbb{R}^{n}$.

For a compact star-shaped (about the origin) *E* in $\mathbb{R}^{n}$, the radial function $\rho_{E}$ of *E*, $\rho_{E}=\rho(E,\cdot)$: $\mathbb {R}^{n}\backslash\{0\}\rightarrow[0,+\infty)$, is defined by (see [1, 2])

$$\rho(E,x)=\max\{\lambda\geq0:\lambda x\in E\},\quad x\in\mathbb {R}^{n}\backslash\{0\}. $$

If $\rho_{E}$ is positive and continuous, then *E* is called a star body.

If $E\in\mathbb{R}^{n}$ is a nonempty subset, the polar set of *E*, $E^{\ast}$, is defined by (see [1, 2])

$$E^{\ast}=\bigl\{ x\in\mathbb{R}^{n}:x\cdot y\leq1, y\in E\bigr\} . $$

From this, it is easy to get that $(E^{\ast})^{\ast}=E$ for all $E\in \mathcal{K}_{o}^{n}$.

From the definition of polar, we know that if $E\in\mathcal{K}_{o}^{n}$, the support and radial function of $E^{\ast}$, the polar body of *E*, have the following relationships (see [1, 2]):

1.1 $$ h\bigl(E^{\ast},\cdot\bigr)=\frac{1}{\rho(E,\cdot)},\qquad \rho \bigl(E^{\ast},\cdot\bigr)=\frac{1}{h(E,\cdot)}. $$

Very recently, Lutwak et al. defined a new concept (i.e., $L_{p}$ dual curvature measures) as follows (see [36]): For $p,q\in\mathbb{R}$, $K\in\mathcal{K}_{o}^{n}$, and $L\in\mathcal{S}_{o}^{n}$, the $L_{p}$ dual curvature measures $\widetilde{C}_{p,q}(K,L,\cdot)$ on $S^{n-1}$ is defined by

1.2 $$ \int_{S^{n-1}}g(v)\,d\widetilde{C}_{p,q}(K,L,v)= \frac{1}{n} \int _{S^{n-1}}g\bigl(\alpha_{K}(u)\bigr)h_{K}^{-p} \bigl(\alpha_{K}(u)\bigr)\rho_{K}^{q}(u)\rho _{L}^{n-q}(u)\,du $$

for each continuous $g:S^{n-1}\rightarrow\mathbb{R}$. Here $\alpha_{K}$ is the radial Gauss map (see [36]).

By (1.2), Lutwak, Yang, and Zhang [36] defined the $(p,q)$-mixed volumes as follows: For $K,L\in\mathcal{K}_{o}^{n}$, $M\in\mathcal{S}_{o}^{n}$, and $p,q\in\mathbb{R}$, the $(p,q)$-mixed volume $\widetilde {V}_{p,q}(K,L,M)$ of $K,L,M$ is defined by

$$\widetilde{V}_{p,q}(K,L,M)= \int_{S^{n-1}}h_{L}^{p}(v)\,d\widetilde {C}_{p,q}(K,M,v). $$

For the $(p,q)$-mixed volumes, the authors [36] gave the following integral formula:

1.3 $$ \widetilde{V}_{p,q}(K,L,M)=\frac{1}{n} \int_{S^{n-1}} \biggl(\frac {h_{L}}{h_{K}} \biggr)^{p}\bigl( \alpha_{K}(u)\bigr)\rho_{K}^{q}(u) \rho_{M}^{n-q}(u)\,du. $$

By (1.3), Lutwak et al. introduced the $L_{p}$ mixed volume for $p\in \mathbb{R}$. For $K,L\in\mathcal{K}_{o}^{n}$ and any real *p*, the $L_{p}$ mixed volume $V_{p}(K,L)$ is given by

$$V_{p}(K,L)=\frac{1}{n} \int_{S^{n-1}}h_{L}^{p}(u)\,dS_{p}(K,u). $$

Here $S_{p}(K, \cdot)$ denotes the $L_{p}$ surface area measure (see [3]). The case of $p\geq1$ is Lutwak’s $L_{p}$ mixed volume (see [3]).

At the same time, for $K,L\in\mathcal{S}_{o}^{n}$ and $q\in\mathbb{R}$, they also defined the *q*th dual mixed volume $\widetilde{V}_{q}(K,L)$ by

$$\widetilde{V}_{q}(K,L)=\frac{1}{n} \int_{S^{n-1}}\rho_{K}^{q}(u)\rho _{L}^{n-q}(u)\,du. $$

In addition, they gave several special cases of $(p,q)$-mixed volume: For $p,q\in\mathbb{R}$, $K,L\in\mathcal{K}_{o}^{n}$, and $M\in\mathcal {S}_{o}^{n}$, then

1.4 $$\begin{aligned} &\widetilde{V}_{p,q}(K,K,K)=V(K)=\frac{1}{n} \int_{S^{n-1}}\rho _{K}^{n}(u)\,du, \end{aligned}$$

1.5 $$\begin{aligned} & \widetilde{V}_{p,q}(K,K,M)=\widetilde{V}_{q}(K,M), \end{aligned}$$

1.6 $$\begin{aligned} & \widetilde{V}_{p,q}(K,L,K)=V_{p}(K,L), \end{aligned}$$

1.7 $$\begin{aligned} & \widetilde{V}_{0,q}(K,L,M)=\widetilde{V}_{q}(K,M), \end{aligned}$$

1.8 $$\begin{aligned} & \widetilde{V}_{p,n}(K,L,M)=V_{p}(K,L). \end{aligned}$$

In this paper, we further study the $(p,q)$-mixed volumes and establish some inequalities including cyclic inequalities, monotonic inequalities, and product inequalities. First, we give a class of cyclic inequalities as follows.

### Theorem 1.1

*Suppose* $p,q,r,s\in\mathbb{R}$ *satisfy* $1\leq p< q< r\leq n$. *If* $K,L\in\mathcal{K}_{o}^{n}$ *and* $M\in\mathcal {S}_{o}^{n}$, *then*

1.9 $$\begin{aligned} \widetilde{V}_{q,s}(K,L,M)^{r-p}\leq\widetilde {V}_{p,s}(K,L,M)^{r-q}\widetilde{V}_{r,s}(K,L,M)^{q-p} \end{aligned}$$

*with equality if and only if* $K,L$, *and* *M* *are dilates*.

### Theorem 1.2

*Suppose* $p,q,r,s\in\mathbb{R}$ *satisfy* $1\leq p< q< r\leq n$. *If* $K,L\in\mathcal{K}_{o}^{n}$ *and* $M\in\mathcal {S}_{o}^{n}$, *then*

1.10 $$\begin{aligned} \widetilde{V}_{s,q}(K,L,M)^{r-p}\leq\widetilde {V}_{s,p}(K,L,M)^{r-q}\widetilde{V}_{s,r}(K,L,M)^{q-p} \end{aligned}$$

*with equality if and only if* $K,L$, *and* *M* *are dilates*.

Then we obtain a type of monotonic inequalities as follows.

### Theorem 1.3

*Suppose* $p,q\in\mathbb{R}$ *satisfy* $1\leq p< q< n$. *If* $K,L\in\mathcal{K}_{o}^{n}$ *and* $M\in\mathcal{S}_{o}^{n}$, *then*

1.11 $$\begin{aligned} \biggl[\frac{\widetilde{V}_{n-p,p}(K,L,M)}{V(K)} \biggr]^{\frac {1}{n-p}}\geq \biggl[ \frac{\widetilde{V}_{n-q,q}(K,L,M)}{V(K)} \biggr]^{\frac{1}{n-q}} \end{aligned}$$

*with equality if and only if* *K* *and* *M* *are dilates*.

### Theorem 1.4

*Suppose* $p,q\in\mathbb{R}$ *satisfy* $1\leq p< q< n$. *If* $K,L\in\mathcal{K}_{o}^{n}$ *and* $M\in\mathcal{S}_{o}^{n}$, *then*

1.12 $$\begin{aligned} \biggl[\frac{\widetilde{V}_{p,p}(K,L,M)}{V(M)} \biggr]^{\frac{1}{p}}\leq \biggl[ \frac{\widetilde{V}_{q,q}(K,L,M)}{V(M)} \biggr]^{\frac{1}{q}} \end{aligned}$$

*with equality if and only if* *K* *and* *M* *are dilates*.

Finally, we set up a type of product inequalities as follows.

### Theorem 1.5

*Suppose* $p>0, q\in\mathbb{R}$. *If* $K,L\in\mathcal{K}_{o}^{n}$ *and* $M\in\mathcal{S}_{o}^{n}$, *then*

1.13 $$\begin{aligned} \widetilde{V}_{p,q}(K,L,M)\widetilde{V}_{p,q} \bigl(K,L^{\ast},M\bigr)\geq\widetilde {V}_{p,q}(K,B,M)^{2} \end{aligned}$$

*with equality if and only if* *L* *is a ball centered at the origin*.

### Theorem 1.6

*Suppose* $p,q\in\mathbb{R}$ *and* $q>n$. *If* $K,L,M\in\mathcal{K}_{o}^{n}$, *then*

1.14 $$\begin{aligned} \widetilde{V}_{p,q}(K,L,M)\widetilde{V}_{p,q} \bigl(K,L,M^{\ast}\bigr)\geq\widetilde {V}_{p,q}(K,L,B)^{2} \end{aligned}$$

*with equality if and only if* *M* *is a ball centered at the origin*.

The proofs of Theorems 1.1–1.6 will be completed in the next section.

## Proofs of theorems

In this part, we give the proofs of Theorems 1.1–1.6.

### Proof of Theorem 1.1

For $p,q,r,s\in\mathbb{R}$, $K,L\in \mathcal{K}_{o}^{n}$, and $M\in\mathcal{S}_{o}^{n}$. Since $1\leq p< q< r\leq n$, then $\frac{r-p}{r-q}>1$. From (1.3) and Hölder’s integral inequality, we get that for $u\in S^{n-1}$

$$\begin{aligned} &\widetilde{V}_{p,s}(K,L,M)^{\frac{r-q}{r-p}}\widetilde {V}_{r,s}(K,L,M)^{\frac{q-p}{r-p}} \\ &\quad = \biggl[\frac{1}{n} \int_{S^{n-1}} \biggl(\frac{h_{L}}{h_{K}} \biggr)^{p}\bigl( \alpha _{K}(u)\bigr)\rho_{K}^{s}(u) \rho_{M}^{n-s}(u)\,du \biggr]^{\frac{r-q}{r-p}} \\ &\qquad {}\cdot \biggl[\frac{1}{n} \int_{S^{n-1}} \biggl(\frac{h_{L}}{h_{K}} \biggr)^{r}\bigl( \alpha _{K}(u)\bigr)\rho_{K}^{s}(u) \rho_{M}^{n-s}(u)\,du \biggr]^{\frac{q-p}{r-p}} \\ &\quad = \biggl[\frac{1}{n} \int_{S^{n-1}} \biggl( \biggl(\frac{h_{L}}{h_{K}} \biggr)^{\frac {p(r-q)}{r-p}}\bigl(\alpha_{K}(u)\bigr)\rho_{K}^{\frac{s(r-q)}{r-p}}(u) \rho_{M}^{\frac{(n-s)(r-q)}{r-p}}(u) \biggr)^{\frac{r-p}{r-q}}\,du \biggr]^{\frac{r-q}{r-p}} \\ &\qquad {}\cdot \biggl[\frac{1}{n} \int_{S^{n-1}} \biggl( \biggl(\frac{h_{L}}{h_{K}} \biggr)^{\frac{r(q-p)}{r-p}}\bigl(\alpha_{K}(u)\bigr)\rho_{K}^{\frac{s(q-p)}{r-p}}(u) \rho_{M}^{\frac{(n-s)(q-p)}{r-p}}(u) \biggr)^{\frac{r-p}{q-p}}\,du \biggr]^{\frac{q-p}{r-p}} \\ &\quad \geq\frac{1}{n} \int_{S^{n-1}} \biggl(\frac{h_{L}}{h_{K}} \biggr)^{q}\bigl( \alpha _{K}(u)\bigr)\rho_{K}^{s}(u) \rho_{M}^{n-s}(u)\,du \\ &\quad =\widetilde{V}_{q,s}(K,L,M), \end{aligned}$$

i.e.,

2.1 $$\begin{aligned} \widetilde{V}_{q,s}(K,L,M)^{r-p}\leq\widetilde {V}_{p,s}(K,L,M)^{r-q}\widetilde{V}_{r,s}(K,L,M)^{q-p}. \end{aligned}$$

This yields (1.9). According to the equality condition of Hölder’s integral inequality, we see that equality holds in (2.1) if and only if $K,L$, and *M* are dilates. □

In (2.1), if $M=K$ or $s=n$, by (1.6) or (1.8), we can get the following result (see [23]).

### Corollary 2.1

*Suppose that* $p,q,r\in\mathbb{R}$ *satisfy* $1\leq p< q< r\leq n$. *If* $K,L\in\mathcal{K}_{o}^{n}$, *then*

$$V_{q}(K,L)^{r-p}\leq V_{p}(K,L)^{r-q}V_{r}(K,L)^{q-p} $$

*with equality if and only if* *K* *and* *L* *are dilates*.

### Proof of Theorem 1.2

For $p,q,r,s\in\mathbb{R}$, $K,L\in \mathcal{K}_{o}^{n}$, and $M\in\mathcal{S}_{o}^{n}$. Since $1\leq p< q< r\leq n$, then $\frac{r-p}{r-q}>1$. From (1.3) and Hölder’s integral inequality, we get that for $u\in S^{n-1}$

$$\begin{aligned} &\widetilde{V}_{s,p}(K,L,M)^{\frac{r-q}{r-p}}\widetilde {V}_{s,r}(K,L,M)^{\frac{q-p}{r-p}} \\ &\quad = \biggl[\frac{1}{n} \int_{S^{n-1}} \biggl(\frac{h_{L}}{h_{K}} \biggr)^{s}\bigl( \alpha _{K}(u)\bigr)\rho_{K}^{p}(u) \rho_{M}^{n-p}(u)\,du \biggr]^{\frac{r-q}{r-p}} \\ &\qquad {}\cdot \biggl[\frac{1}{n} \int_{S^{n-1}} \biggl(\frac{h_{L}}{h_{K}} \biggr)^{s}\bigl( \alpha _{K}(u)\bigr)\rho_{K}^{r}(u) \rho_{M}^{n-r}(u)\,du \biggr]^{\frac{q-p}{r-p}} \\ &\quad = \biggl[\frac{1}{n} \int_{S^{n-1}} \biggl( \biggl(\frac{h_{L}}{h_{K}} \biggr)^{\frac {s(r-q)}{r-p}}\bigl(\alpha_{K}(u)\bigr)\rho_{K}^{\frac{p(r-q)}{r-p}}(u) \rho_{M}^{\frac{(n-p)(r-q)}{r-p}}(u) \biggr)^{\frac{r-p}{r-q}}\,du \biggr]^{\frac{r-q}{r-p}} \\ &\qquad {}\cdot \biggl[\frac{1}{n} \int_{S^{n-1}} \biggl( \biggl(\frac{h_{L}}{h_{K}} \biggr)^{\frac{s(q-p)}{r-p}}\bigl(\alpha_{K}(u)\bigr)\rho_{K}^{\frac{r(q-p)}{r-p}}(u) \rho_{M}^{\frac{(n-r)(q-p)}{r-p}}(u) \biggr)^{\frac{r-p}{q-p}}\,du \biggr]^{\frac{q-p}{r-p}} \\ &\quad \geq\frac{1}{n} \int_{S^{n-1}} \biggl(\frac{h_{L}}{h_{K}} \biggr)^{s}\bigl( \alpha _{K}(u)\bigr)\rho_{K}^{q}(u) \rho_{M}^{n-q}(u)\,du \\ &\quad =\widetilde{V}_{s,q}(K,L,M). \end{aligned}$$

Thus, we get

2.2 $$\begin{aligned} \widetilde{V}_{s,q}(K,L,M)^{r-p}\leq\widetilde {V}_{s,p}(K,L,M)^{r-q}\widetilde{V}_{s,r}(K,L,M)^{q-p}. \end{aligned}$$

This yields (1.10). According to the equality condition of Hölder’s integral inequality, we see that equality holds in (2.2) if and only if $K,L$, and *M* are dilates. □

Combined with (1.5) and (1.7), taking $L=K$ or $s=0$ in (2.2), we obtain the following corollary.

### Corollary 2.2

*Suppose* $p,q,r\in\mathbb{R}$ *satisfy* $1\leq p< q< r\leq n$. *If* $K\in\mathcal{K}_{o}^{n}$ *and* $M\in\mathcal{S}_{o}^{n}$, *then*

$$\widetilde{V}_{q}(K,M)^{r-p}\leq\widetilde{V}_{p}(K,M)^{r-q} \widetilde {V}_{r}(K,M)^{q-p} $$

*with equality if and only if* *K* *and* *M* *are dilates*.

### Proof of Theorem 1.3

For $p,q\in\mathbb{R}$, $K,L\in \mathcal{K}_{o}^{n}$, and $M\in\mathcal{S}_{o}^{n}$. Since $1\leq p< q$, then $\frac{n-q}{n-p}<1$. From (1.3), (1.4), and Hölder’s integral inequality, we obtain that for $u\in S^{n-1}$

$$\begin{aligned} &\widetilde{V}_{n-q,q}(K,L,M)^{\frac{n-p}{n-q}}V(K)^{\frac{p-q}{n-q}} \\ &\quad = \biggl[\frac{1}{n} \int_{S^{n-1}} \biggl(\frac{h_{L}}{h_{K}} \biggr)^{n-q} \bigl(\alpha _{K}(u)\bigr)\rho_{K}^{q}(u) \rho_{M}^{n-q}(u)\,du \biggr]^{\frac{n-p}{n-q}} \cdot \biggl[ \frac{1}{n} \int_{S^{n-1}}\rho_{K}^{n}(u)\,du \biggr]^{\frac {p-q}{n-q}} \\ &\quad = \biggl[\frac{1}{n} \int_{S^{n-1}} \biggl( \biggl(\frac{h_{L}}{h_{K}} \biggr)^{n-p}\bigl(\alpha_{K}(u)\bigr)\rho_{K}^{\frac{q(n-p)}{n-q}}(u) \rho_{M}^{n-p}(u) \biggr)^{\frac{n-q}{n-p}}\,du \biggr]^{\frac{n-p}{n-q}} \\ &\qquad {}\cdot \biggl[\frac{1}{n} \int_{S^{n-1}} \bigl(\rho_{K}^{\frac {n(p-q)}{n-q}}(u) \bigr)^{\frac{n-q}{p-q}}\,du \biggr]^{\frac{p-q}{n-q}} \\ &\quad \leq\frac{1}{n} \int_{S^{n-1}} \biggl(\frac{h_{L}}{h_{K}} \biggr)^{n-p} \bigl(\alpha _{K}(u)\bigr)\rho_{K}^{p}(u) \rho_{M}^{n-p}(u)\,du \\ &\quad =\widetilde{V}_{n-p,p}(K,L,M), \end{aligned}$$

i.e.,

2.3 $$\begin{aligned} \biggl[\frac{\widetilde{V}_{n-p,p}(K,L,M)}{V(K)} \biggr]^{\frac {1}{n-p}}\geq \biggl[ \frac{\widetilde{V}_{n-q,q}(K,L,M)}{V(K)} \biggr]^{\frac{1}{n-q}}. \end{aligned}$$

This gives (1.11). According to the equality condition of Hölder’s integral inequality, we know that equality holds in (2.3) if and only if *K* and *M* are dilates. □

### Proof of Theorem 1.4

For $p,q\in\mathbb{R}$, $K,L\in \mathcal{K}_{o}^{n}$, and $M\in\mathcal{S}_{o}^{n}$. Since $1\leq p< q$, then $\frac{q}{p}>1$. From (1.3), (1.4), and Hölder’s integral inequality, we obtain that for $u\in S^{n-1}$

$$\begin{aligned} &\widetilde{V}_{q,q}(K,L,M)^{\frac{p}{q}}V(M)^{\frac{q-p}{q}} \\ &\quad = \biggl[\frac{1}{n} \int_{S^{n-1}} \biggl(\frac{h_{L}}{h_{K}} \biggr)^{q} \bigl(\alpha _{K}(u)\bigr)\rho_{K}^{q}(u) \rho_{M}^{n-q}(u)\,du \biggr]^{\frac{p}{q}} \cdot \biggl[ \frac{1}{n} \int_{S^{n-1}}\rho_{M}^{n}(u)\,du \biggr]^{\frac {q-p}{q}} \\ &\quad = \biggl[\frac{1}{n} \int_{S^{n-1}} \biggl( \biggl(\frac{h_{L}}{h_{K}} \biggr)^{p}\bigl(\alpha_{K}(u)\bigr)\rho_{K}^{p}(u) \rho_{M}^{\frac{p(n-q)}{q}}(u) \biggr)^{\frac {q}{p}}\,du \biggr]^{\frac{p}{q}} \\ &\qquad {}\cdot \biggl[\frac{1}{n} \int_{S^{n-1}} \bigl(\rho_{M}^{\frac {n(q-p)}{q}}(u) \bigr)^{\frac{q}{q-p}}\,du \biggr]^{\frac{q-p}{q}} \\ &\quad \geq\frac{1}{n} \int_{S^{n-1}} \biggl(\frac{h_{L}}{h_{K}} \biggr)^{p}\bigl( \alpha _{K}(u)\bigr)\rho_{K}^{p}(u) \rho_{M}^{n-p}(u)\,du \\ &\quad =\widetilde{V}_{p,p}(K,L,M), \end{aligned}$$

i.e.,

2.4 $$\begin{aligned} \biggl[\frac{\widetilde{V}_{p,p}(K,L,M)}{V(M)} \biggr]^{\frac{1}{p}}\leq \biggl[ \frac{\widetilde{V}_{q,q}(K,L,M)}{V(M)} \biggr]^{\frac{1}{q}}. \end{aligned}$$

This gives (1.12). According to the equality condition of Hölder’s integral inequality, we know that equality holds in (2.4) if and only if *K* and *M* are dilates. □

We can get the following corollary by (1.5) and (1.7) in (2.4).

### Corollary 2.3

*Suppose* $p,q\in\mathbb{R}$ *satisfy* $1\leq p< q$. *If* $K\in\mathcal{K}_{o}^{n}$ *and* $M\in\mathcal{S}_{o}^{n}$, *then*

$$\biggl[\frac{\widetilde{V}_{p}(K,M)}{V(M)} \biggr]^{\frac{1}{p}}\leq \biggl[\frac{\widetilde{V}_{q}(K,M)}{V(M)} \biggr]^{\frac{1}{q}} $$

*with equality if and only if* *K* *and* *M* *are dilates*.

### Proof of Theorem 1.5

For $p>0, q\in\mathbb{R}$, $K,L\in \mathcal{K}_{o}^{n}$, and $M\in\mathcal{S}_{o}^{n}$. From the definitions of support function and radial function, we know

2.5 $$\begin{aligned} \rho_{L^{\ast}}(u)\leq h_{L^{\ast}}(u) \end{aligned}$$

with equality if and only if *L* is a ball centered at the origin.

From (1.3), (1.1), (2.5), and Cauchy’s integral inequality, and noticing that $h(B,\cdot)=1$, we have

$$\begin{aligned} &\widetilde{V}_{p,q}(K,L,M)^{\frac{1}{2}}\widetilde{V}_{p,q} \bigl(K,L^{\ast},M\bigr)^{\frac{1}{2}} \\ &\quad = \biggl[\frac{1}{n} \int_{S^{n-1}} \biggl(\frac{h_{L}}{h_{K}} \biggr)^{p}\bigl( \alpha _{K}(u)\bigr)\rho_{K}^{q}(u) \rho_{M}^{n-q}(u)\,du \biggr]^{\frac{1}{2}} \\ &\qquad {}\cdot \biggl[\frac{1}{n} \int_{S^{n-1}} \biggl(\frac{h_{L^{\ast}}}{h_{K}} \biggr)^{p}\bigl( \alpha_{K}(u)\bigr)\rho_{K}^{q}(u) \rho_{M}^{n-q}(u)\,du \biggr]^{\frac{1}{2}} \\ &\quad = \biggl[\frac{1}{n} \int_{S^{n-1}}\rho_{L^{\ast}}^{-p}\bigl(\alpha _{K}(u)\bigr)h_{K}^{-p}\bigl( \alpha_{K}(u)\bigr)\rho_{K}^{q}(u) \rho_{M}^{n-q}(u)\,du \biggr]^{\frac {1}{2}} \\ &\qquad {}\cdot \biggl[\frac{1}{n} \int_{S^{n-1}}h_{L^{\ast}}^{p}\bigl(\alpha _{K}(u)\bigr)h_{K}^{-p}\bigl( \alpha_{K}(u)\bigr)\rho_{K}^{q}(u) \rho_{M}^{n-q}(u)\,du \biggr]^{\frac {1}{2}} \\ &\quad \geq\frac{1}{n} \int_{S^{n-1}} \biggl(\frac{h_{L^{\ast}}}{\rho_{L^{\ast}}} \biggr)^{\frac{p}{2}} \bigl(\alpha_{K}(u)\bigr)h_{K}^{-p}\bigl( \alpha_{K}(u)\bigr)\rho_{K}^{q}(u)\rho _{M}^{n-q}(u)\,du \\ &\quad \geq\frac{1}{n} \int_{S^{n-1}}h_{K}^{-p}\bigl( \alpha_{K}(u)\bigr)\rho_{K}^{q}(u)\rho _{M}^{n-q}(u)\,du \\ &\quad =\frac{1}{n} \int_{S^{n-1}} \biggl(\frac{h_{B}}{h_{K}} \biggr)^{p}\bigl( \alpha_{K}(u)\bigr)\rho _{K}^{q}(u) \rho_{M}^{n-q}(u)\,du \\ &\quad =\widetilde{V}_{p,q}(K,B,M), \end{aligned}$$

i.e.,

2.6 $$\begin{aligned} \widetilde{V}_{p,q}(K,L,M)\widetilde{V}_{p,q} \bigl(K,L^{\ast},M\bigr)\geq\widetilde {V}_{p,q}(K,B,M)^{2}. \end{aligned}$$

Obviously, equality holds in (2.6) if and only if *L* is a ball centered at the origin. □

If we take $M=K$ or $q=n$ in (2.6) and associate with (1.6) and (1.8), the following corollary can be obtained (see [37]).

### Corollary 2.4

*Suppose* $p>0$. *If* $K, L\in\mathcal{K}_{o}^{n}$, *then*

$$V_{p}(K,L)V_{p}\bigl(K,L^{\ast}\bigr)\geq V_{p}(K,B)^{2} $$

*with equality if and only if* *L* *is a ball centered at the origin*.

### Proof of Theorem 1.6

For $p,q\in\mathbb{R}$ and $q>n$, $K,L,M\in\mathcal{K}_{o}^{n}$. From the definitions of support function and radial function, we know

2.7 $$\begin{aligned} \rho_{M}(u)\leq h_{M}(u) \end{aligned}$$

with equality if and only if *M* is a ball centered at the origin.

From (1.3), (1.1), (2.7), and Cauchy’s integral inequality, and together with $\rho(B,\cdot)=1$, we obtain

$$\begin{aligned} &\widetilde{V}_{p,q}(K,L,M)\widetilde{V}_{p,q} \bigl(K,L,M^{\ast}\bigr) \\ &\quad = \biggl[\frac{1}{n} \int_{S^{n-1}} \biggl(\frac{h_{L}}{h_{K}} \biggr)^{p}\bigl( \alpha _{K}(u)\bigr)\rho_{K}^{q}(u) \rho_{M}^{n-q}(u)\,du \biggr] \\ &\qquad {}\cdot \biggl[\frac{1}{n} \int_{S^{n-1}} \biggl(\frac{h_{L}}{h_{K}} \biggr)^{p}\bigl( \alpha_{K}(u)\bigr)\rho_{K}^{q}(u) \rho_{M^{\ast}}^{n-q}(u)\,du \biggr] \\ &\quad = \biggl[\frac{1}{n} \int_{S^{n-1}} \biggl(\frac{h_{L}}{h_{K}} \biggr)^{p}\bigl( \alpha _{K}(u)\bigr)\rho_{K}^{q}(u) \rho_{M}^{n-q}(u)\,du \biggr] \\ &\qquad {}\cdot \biggl[\frac{1}{n} \int_{S^{n-1}} \biggl(\frac{h_{L}}{h_{K}} \biggr)^{p}\bigl( \alpha_{K}(u)\bigr)\rho_{K}^{q}(u)h_{M}^{q-n}(u)\,du \biggr] \\ &\quad \geq \biggl[\frac{1}{n} \int_{S^{n-1}} \biggl(\frac{h_{L}}{h_{K}} \biggr)^{p}\bigl( \alpha _{K}(u)\bigr)\rho_{K}^{q}(u) \biggl( \frac{h_{M}}{\rho_{M}} \biggr)^{\frac {q-n}{2}}(u)\,du \biggr]^{2} \\ &\quad \geq \biggl[\frac{1}{n} \int_{S^{n-1}} \biggl(\frac{h_{L}}{h_{K}} \biggr)^{p}\bigl( \alpha_{K}(u)\bigr)\rho_{K}^{q}(u)\,du \biggr]^{2} \\ &\quad = \biggl[\frac{1}{n} \int_{S^{n-1}} \biggl(\frac{h_{L}}{h_{K}} \biggr)^{p}\bigl( \alpha _{K}(u)\bigr)\rho_{K}^{q}(u) \rho_{B}^{n-q}(u)\,du \biggr]^{2} \\ &\quad =\widetilde{V}_{p,q}(K,L,B)^{2}, \end{aligned}$$

This gives (1.14). Obviously, according to the equality of (2.7), we know that equality holds in (1.14) if and only if *M* is a ball centered at the origin. □

By (1.5) and (1.7), taking $L=K$ or $p=0$ in Theorem 1.6, we also obtain the following corollary.

### Corollary 2.5

*Suppose* $q\in\mathbb{R}$ *and* $q>n$. *If* $K,M\in\mathcal{K}_{o}^{n}$, *then*

$$\widetilde{V}_{q}(K,M)\widetilde{V}_{q} \bigl(K,M^{\ast}\bigr)\geq\widetilde {V}_{q}(K,B)^{2} $$

*with equality if and only if* *M* *is a ball centered at the origin*.
